# Establishing Syndromic Surveillance of Acute Coronary Syndrome, Myocardial Infarction, and Stroke: Registry Study Based on Routine Data From German Emergency Departments

**DOI:** 10.2196/66218

**Published:** 2025-02-25

**Authors:** Madlen Schranz, Mirjam Rupprecht, Annette Aigner, Leo Benning, Carmen Schlump, Nesrine Charfeddine, Michaela Diercke, Linus Grabenhenrich, Alexander Ullrich, Hannelore Neuhauser, Birga Maier, AKTIN Research Group, Tobias Hofmann, Felix Patricius Hans, Sabine Blaschke

**Affiliations:** 1 Department for Infectious Disease Epidemiology, Robert Koch Institute, Berlin, Germany; 2 Institute of Public Health, Charité – Universitätsmedizin Berlin, corporate member of Freie Universiät Berlin and Humboldt-Universität zu Berlin, Berlin, Germany; 3 Institute of Biometry and Clinical Epidemiology, Charité – Universitätsmedizin Berlin, corporate member of Freie Universiät Berlin and Humboldt-Universität zu Berlin, Berlin, Germany; 4 Center for Stroke Research Berlin, Charité - Universitätsmedizin Berlin, corporate member of Freie Universiät Berlin and Humboldt-Universität zu Berlin, Berlin, Germany; 5 University Emergency Department, Medical Center, Faculty of Medicine, University of Freiburg, Freiburg, Germany; 6 Department for Method Development, Research Infrastructure and Information Technology, Robert Koch Institute, Berlin, Germany; 7 Department for Epidemiology and Health Monitoring, Robert Koch Institute, Berlin, Germany; 8 DZHK (German Centre for Cardiovascular Research), Berlin, Germany; 9 Emergency Department, University Medical Center Göttingen, Göttingen, Germany

**Keywords:** emergency medicine, routinely collected health data, public health surveillance, syndromic surveillance, acute coronary syndrome, myocardial infarction, stroke, routine data, Germany, emergency department, accuracy, syndrome, diagnosis, public health, health surveillance

## Abstract

**Background:**

Emergency department (ED) routine data offer a unique opportunity for syndromic surveillance of communicable and noncommunicable diseases (NCDs). In 2020, the Robert Koch Institute established a syndromic surveillance system using ED data from the AKTIN registry. The system provides daily insights into ED utilization for infectious diseases. Adding NCD indicators to the surveillance is of great public health importance, especially during acute events, where timely monitoring enables targeted public health responses and communication.

**Objective:**

This study aimed to develop and validate syndrome definitions for the NCD indicators of acute coronary syndrome (ACS), myocardial infarction (MI), and stroke (STR).

**Methods:**

First, syndrome definitions were developed with clinical experts combining ED diagnosis, chief complaints, diagnostic certainty, and discharge information. Then, using the multicenter retrospective routine ED data provided by the AKTIN registry, we conducted internal validation by linking ED cases fulfilling the syndrome definition with the hospital discharge diagnoses and calculating sensitivity, specificity, and accuracy. Lastly, external validation comprised the comparison of the ED cases fulfilling the syndrome definition with the federal German hospital diagnosis statistic. Ratios comparing the relative number of cases for all syndrome definitions were calculated and stratified by age and sex.

**Results:**

We analyzed data from 9 EDs, totaling 704,797 attendances from January 1, 2019, to March 5, 2021. Syndrome definitions were based on *ICD-10* (International Statistical Classification of Diseases and Related Health Problems 10th Revision-German Modification) diagnoses, chief complaints, and discharge information. We identified 4.3% of all cases as ACS, 0.6% as MI, and 3.2% as STR. Patients with ACS and MI were more likely to be male (58.3% and 64.7%), compared to the overall attendances (52.7%). For all syndrome definitions, the prevalence was higher in the older age groups (60‐79 years and >80 years), and the highest proportions of cases were assigned an urgency level (3=urgent or 2=very urgent). The internal validation showed accuracy and specificity levels above 96% for all syndrome definitions. The sensitivity was 85.3% for ACS, 56.6% for MI, and 80.5% for STR. The external validation showed high levels of correspondence between the ED data and the German hospital statistics, with most ratios ranging around 1, indicating congruence, particularly in older age groups. The highest differences were noted in younger age groups, with the highest ratios in women aged between 20 and 39 years (4.57 for MI and 4.17 for ACS).

**Conclusions:**

We developed NCD indicators for ACS, MI, and STR that showed high levels of internal and external validity. The integration of these indicators into the syndromic surveillance system for EDs could enable daily monitoring of NCD patterns and trends to enhance timely public health surveillance in Germany.

## Introduction

Syndromic surveillance using routine data from emergency departments (EDs) has been proven a valuable tool to assess trends in ED utilization and monitor disease occurrence over time [1]. The Robert Koch Institute (RKI), a federal agency and research institute responsible for disease control and prevention in Germany, has recognized the practical benefits of this approach. At the RKI, an ED syndromic surveillance system has been established in 2020, using daily routine data from the German Emergency Department Data Registry AKTIN [2]. Currently, 58 EDs in 12 German federal states voluntarily provide data for research and surveillance purposes. The AKTIN registry accounts for EDs of all 3 levels of care according to the German system of staged emergency care, which divides EDs into extended, comprehensive, or basic emergency care facilities. For each level, the Federal Joint Committee, a key stakeholder overseeing and providing legal guidance for healthcare delivery in the German health care system, defined specific requirements like necessary clinical departments, intensive care capacities, and medical equipment [3]. The ED surveillance provides a timely view of the utilization of EDs through a digital dashboard that is updated daily. This helps to identify trends and patterns, thereby aiding in the formulation of effective public health strategies. It currently monitors infectious diseases, specifically respiratory and gastrointestinal infections [4]. However, these health events only represent a small proportion of all attendances to German EDs. Noncommunicable diseases (NCDs), such as cardiovascular and neurological diseases, are nowadays among the most relevant.

EDs in Germany often serve as an initial point of contact with the health care system for the population, especially for severe and acute health events and medical conditions. This aspect offers a potential time advantage in detecting health events of interest and enabling earlier identification and response compared to other data sources. Yet, Germany currently does not have a surveillance system providing timely information on NCDs, which could especially be important during acute events and situations that impact health at a population level. Evidence suggests, for example, that extreme weather events may lead to an increase in cardiovascular disease and acute myocardial infarction (MI) [5]. A real-time syndromic surveillance system would enable health authorities to rapidly detect these changes and respond with tailored interventions, such as issuing public health warnings to the population. Previous studies have also found that mass gatherings and large sporting events could lead to an increase in cases of cardiovascular disease [6]. Timely surveillance can assist public health authorities in their ad-hoc risk communication and reallocation of emergency care resources. A recent example is the COVID-19 pandemic, which significantly impacted ED attendance, both in Germany [7] and globally [8,9]. A reduction was not only visible in overall attendance but also in medical emergencies like MIs and stroke (STR) [10]. While the reasons for this reduction cannot be causally explained with certainty, there is an ongoing debate about whether patients might have delayed their emergency treatment or decided to completely omit treatment due to fear of infection with SARS-CoV-2. Another possible explanation could have been an actual reduction in the number of cases in the population resulting from behavioral changes during lockdown periods [11]. Continuous and timely monitoring of these changes will be crucial in the future to understand trends and facilitate the early detection of public health emergencies, guide public health interventions, and support the implementation of evidence-based policies.

Expanding the daily ED surveillance at the RKI to include NCD-related indicators could enhance public health efforts by providing timely data on these high-burden health events, thereby supporting strategies aimed at minimizing their impact. However, routine data from EDs are not yet collected for research purposes. Consequently, ED-specific differences in the documentation of medical data can affect the data quality. Therefore, a significant challenge when implementing new indicators into an established surveillance system lies in determining the ability of specific syndrome definitions to sufficiently detect cases of the respective indicator. This study therefore aimed to develop and validate the syndrome definitions for the monitoring of acute coronary syndrome (ACS), MI, and STR, and to assess their potential for a future integration into the RKI’s routine ED surveillance.

## Methods

### Ethical Considerations

All individual patient data were obtained through a data usage request with the AKTIN Emergency Department Data Registry (ID 2022‐001). The AKTIN Emergency Department Data Registry received positive ethics votes for using the ED and hospital discharge data from the Otto von Guericke University Magdeburg ethics committee (160/15, 52/21).

Individual informed patient consent was not required and not feasible in this case, as regulated within the EU General Data Protection Regulation (GDPR) and the German Federal Data Protection Act. Art. 89 GDPR, Art. 9 (2) GDPR, and §22 German Federal Data Protection Act. They regulate data usage for the purpose of research that is of public interest, without the need for informed consent. Compliance with those regulations is addressed in AKTIN’s data protection concept [12]. All of the research described in this manuscript was performed in accordance with the Declaration of Helsinki.

Case-based ED data were provided as an anonymized dataset using a prespecified data model for surveillance and research purposes (Notaufnahme-Kerndatensatz (NoKeDa) [13]). The use of routine ED data according to the NoKeDa data format was assessed and approved by the RKI’s data protection officer.

### Data Sources and Study Population

We used routinely collected data from EDs provided by the AKTIN registry [2] (Project ID 2022‐001). The ED data included information on the patient (age group and sex) and administrative information (ED, date and time of admission, and disposition information). Furthermore, it contained the following information regarding the health status: urgency level coded as per the Manchester Triage System [14] or Emergency Severity Index [15], chief complaint according to the CEDIS-PCL (Canadian Emergency Department Information System–Presenting Complaint List [16]), and one or more diagnoses coded as ICD-10-GM (further referred to as “ED diagnoses,” [17]). All ED data were provided to the RKI as anonymized datasets; multiple visits of individual patients could not be identified.

For validation purposes, we used 2 additional datasets: Through the AKTIN registry, inpatient care data were available for 15 of the EDs in case of inpatient treatment following the ED visit. It included ICD-10-GM discharge diagnoses (further referred to as “hospital discharge diagnoses”), as well as information on diagnosis certainty and whether they were labeled as the “main diagnosis.” The information was routinely collected during inpatient treatment according to the German social laws regulating the billing of inpatient services and obliging hospitals to transmit billing data to a federal agency (§ 21 Krankenhausentgeltgesetz, KHEntgG). Inpatient care data in this case can be directly linked to the ED data by a common attendance-specific identifier in both datasets. Additionally, we used publicly available data from the federal hospital diagnosis statistic, published by the German Federal Statistical Office [18]. Those data are aggregated yearly and include all diagnoses coded in German hospitals, as well as demographic information like age and sex. Given the aggregated nature of the data, linkage to the ED data is not possible in this case.

We included all EDs in the study that provided continuous data between January 1, 2019 and May 3, 2021. Continuous data availability was defined as providing a minimum of one ED attendance with either an ED diagnosis or chief complaint and one hospital discharge diagnosis for each day of the study period. Beyond that, every attendance that was documented in the selected EDs within our study period was included in our analysis.

### Syndrome Definitions

Syndrome definitions were developed in an iterative process, consulting clinical experts from the participating EDs, epidemiological researchers in the field of the respective disease group, and surveillance experts. After considering the setting-specific use of codes for the indicators of interest, we created syndrome definitions to identify attendances with ACS or STR. As a subgroup of ACS, we additionally created a definition for attendances with a suspected MI. Syndrome definitions were based on ED diagnoses, chief complaints, age, and disposition information.

### Statistical Analysis

We conducted descriptive analyses by calculating absolute and relative case numbers. We stratified the results by age, sex, and triage level. The validation of syndrome definitions was performed in 2 steps.

First, we conducted an internal validation by comparing ED cases with their respective hospital discharge diagnosis. Therefore, we linked ED data with inpatient care data on a case-based level, keeping only those attendances present in both datasets (ie, ED patients who have been admitted to the hospital). For each case of ACS, MI, and STR identified in the ED by the syndrome definitions, we looked at the 10 most common hospital discharge diagnoses coded for those cases. We then calculated sensitivity, specificity, and accuracy for all 3 syndrome definitions, along with 95% CI by using each ED case’s hospital discharge diagnosis as a reference standard. We defined the gold standard for the validation of the ED syndromes based on the following criteria: for ACS, cases with the ICD-10 hospital discharge diagnoses I20 or I21; for MI, cases with discharge diagnosis of I21; and for STR, discharge diagnoses of I60, I61, I63, or I64 were considered as true positives. Additionally, to assess the validity of the syndrome definitions over time, we plotted the time series of the ED cases identified by the syndrome definition against those with matching diagnosis in the hospital discharge data, using a 7-day moving average for both.

Second, for external validation, we used data from the hospital diagnosis statistic. As those data were only available aggregated yearly, we restricted our ED dataset to the fully available years 2019 and 2020. We calculated the relative number of ACS, MI, and STR cases identified in the ED by the syndrome definitions stratified by age and sex, as well as the relative number of cases with the same set of diagnoses retrieved from the hospital diagnosis statistic. We calculated ratios with respective 95% CIs comparing ED cases to hospital diagnosis statistics.

All analyses were performed using R (version 4.2.2) [19] and the packages *tidyverse* [20], *table 1 *[21]*, ggpubr* [22]*, xlsx* [23]*, epiR* [24]*, and binom* [25].

## Results

Out of 24 EDs that generally provided data through the AKTIN registry, 8 EDs were excluded because they did not deliver data for the complete study period due to their recent enrollment into the AKTIN registry. Additionally, we excluded 3 EDs for insufficient data quality (inconsistent and missing data) and 4 EDs for not providing the separately collected inpatient care data, resulting in 9 EDs that met our inclusion criteria. The included EDs were located in the federal states of Baden-Württemberg, Bavaria, Lower Saxony, and Saxony-Anhalt. In total, 2 EDs accounted for extended emergency care and 7 for comprehensive emergency care facilities. The mean weekly attendances ranged from 204 to 1491 across the included EDs, resulting in a final sample of 704,797 attendances between January 1, 2019, and March 5, 2021.

### Syndrome Definitions

#### Acute Coronary Syndrome (ACS)

**Figure 1.**
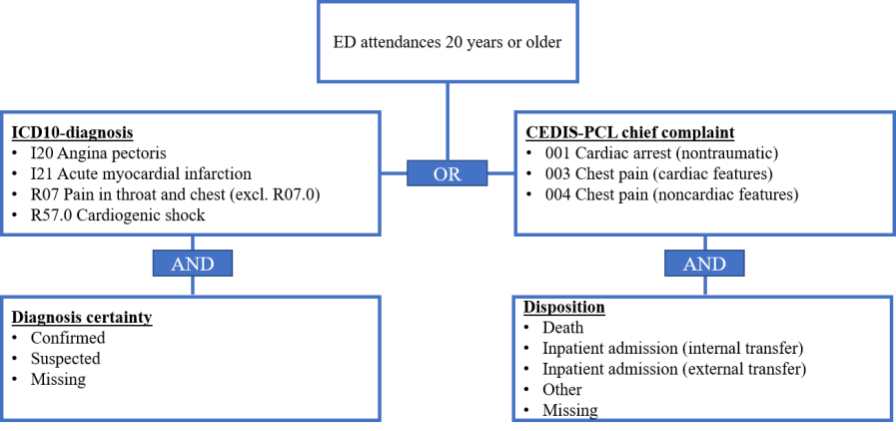
Final syndrome definition for acute coronary syndrome (ACS). ED: emergency department.

For ACS, the ICD-10 ED diagnoses I20, I21, R07 (excluding R07.0), and R57.0 were considered as relevant for the definition, including only those with certainty levels “confirmed,” “suspected,” or diagnoses without a certainty level. Attendances without an ED diagnosis were still considered ACS cases if they received one of the following CEDIS-PCL chief complaints: “001—Cardiac arrest (nontraumatic),” “003—Chest pain (cardiac features),” or “004—Chest pain (noncardiac features).” Since chief complaints are generally less specific compared to diagnoses, we only considered them in combination with one of the following disposition values: “inpatient admission,” “death,” “other value,” or with no disposition information documented. Assuming that ACS generally requires further inpatient treatment, this ensured that we excluded cases discharged home. All attendances had to be at least 20 years of age to be labeled as a case, assuming that ACS is usually not common in children and young adults (Figure 1).

#### Myocardial Infarction (MI)

**Figure 2.**
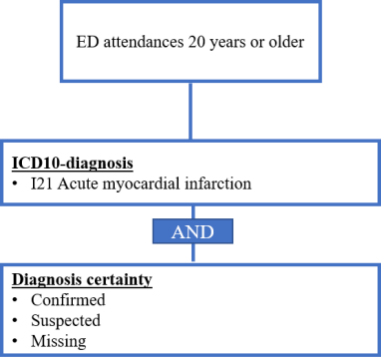
Final syndrome definition for myocardial infarction (MI). ED: emergency department.

For MI, all patients had to be at least 20 years of age and diagnosed with a confirmed or suspected ICD-10 diagnosis I21 in the ED. Diagnoses without certainty information were considered as well (Figure 2).

#### Stroke (STR)

**Figure 3.**
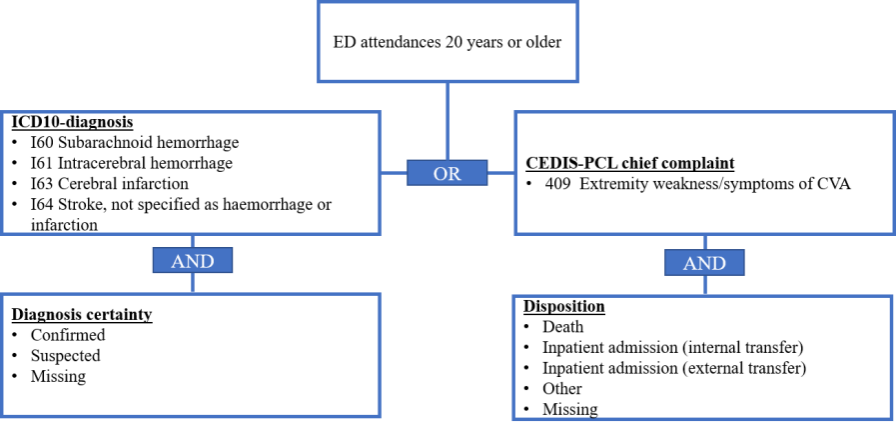
Final syndrome definition for stroke (STR). ED: emergency department; CEDIS-PCL: Canadian Emergency Department Information System–Presenting Complaint List; CVA: cerebrovascular accident.

All attendances receiving a STR-related ICD-10 ED diagnosis (I60, I61, I63, or I64) with certainty levels “confirmed,” “suspected,” or diagnoses without a certainty level were labeled as cases. The catalog lists several CEDIS-PCL chief complaints that code neurological complaints potentially associated with STR (401‐410, except 407 and 411). However, as discussed with clinical experts, the only CEDIS-PCL chief complaint specific enough to identify cases of STR was decided to be “409 - Extremity weakness/symptoms of CVA.” Therefore, in addition to the ICD-10 codes mentioned earlier, attendances with the CEDIS-PCL chief complaint 409 were labeled as a case if they were hospitalized, died, had no disposition information, or had the value “other” documented. All attendances needed to be at least 20 years old to be considered by the syndrome definition (Figure 3) .

### Descriptive Analyses

Overall, we identified 4.3% (n=30,217) of all attendances as cases of ACS. In total, 4,155 were explicitly identified as MI cases, representing a proportion of 0.6% of all attendances. Based on the syndrome definition, we could identify 22,642 cases or 3.2% of attendances as STR cases (Table 1).

**Table 1.** Baseline table of all ED attendances by sex, age, and triage level, as well as stratified by the syndrome definitions acute coronary syndrome (ACS), myocardial infarction (MI), and stroke (STR).

	ACS	MI	STR	All attendances
	n=30,217 (100%)	n=4155 (100%)	n=22,642 (100%)	n=704,797 (100%)
Sex				
Female	12,587 (41.7%)	1465 (35.3%)	10,886 (48.1%)	333,392 (47.3%)
Male	17,630 (58.3%)	2690 (64.7%)	11,756 (51.9%)	371,405 (52.7%)
Age, years				
0‐19	0 (0%)	0 (0%)	0 (0%)	77,346 (11.0%)
20‐39	4543 (15.0%)	177 (4.3%)	900 (4.0%)	172,443 (24.5%)
40‐59	8244 (27.3%)	1023 (24.6%)	3862 (17.1%)	159,474 (22.6%)
60‐79	11,233 (37.2%)	1901 (45.8%)	9835 (43.4%)	176,233 (25.0%)
>80	6197 (20.5%)	1054 (25.4%)	8045 (35.5%)	119,301 (16.9%)
Triage level				
1=immediate	666 (2.2%)	146 (3.5%)	1256 (5.5%)	8669 (1.2%)
2=very urgent	8878 (29.4%)	1583 (38.1%)	9860 (43.5%)	94,107 (13.4%)
3=urgent	10,697 (35.4%)	1331 (32.0%)	5638 (24.9%)	203,535 (28.9%)
4=standard	4228 (14.0%)	327 (7.9%)	1782 (7.9%)	270,973 (38.4%)
5=non-urgent	208 (0.7%)	14 (0.3%)	175 (0.8%)	31,534 (4.5%)
Missing	5540 (18.3%)	754 (18.1%)	3931 (17.4%)	95,979 (13.6%)

When examining the distribution of sex, age, and triage level, slight differences were observed compared to overall attendance. The proportion of males was higher, at 58.3% for ACS and 64.7% for MI, compared to 52.7% for all attendances. All 3 indicators showed higher prevalence among the elderly. The age group of 60‐79 years accounted for 37.2% of ACS cases, 45.8% of MI cases, and 43.4% of STR cases, compared to 25.0% of the overall attendances. We observed a similar pattern for the recorded triage level. The largest group among overall attendance was triage level 4, accounting for 38.5%. In comparison, ACS cases were primarily assigned an urgency level 3—urgent. For MI and STR, the largest proportion of cases was triaged with level 2—very urgent, with 38.1% for MI and 43.6% for STR.

### Internal Validation

For the internal validation, we included only ED attendances that could be linked to hospital discharge diagnoses, resulting in a total of 305,469 or 43.3% of all available ED attendances. In this linked dataset, according to our syndrome definitions, we identified 21,786 cases of ACS, 3711 cases of MI, and 18,428 cases of STR. The distribution of sex, age, and triage level stayed virtually the same compared to all available ED cases. We observed a slightly lower proportion of cases in the youngest age group while slightly more cases were triaged with a higher urgency (Table S1 in Multimedia Appendix 1).

The most common ICD-10 hospital discharge diagnosis groups associated with ED attendance identified by the ACS syndrome definition were R07 (19.7 %), I21 (17.6 %), and I20 (13.3 %) (Table S2 in Multimedia Appendix 1). For the MI syndrome definition, most cases received an ICD-10 hospital discharge diagnosis belonging to the group I21 (70.3 %), while the remaining were distributed across 397 different diagnosis groups (Table S3 in Multimedia Appendix 1). Half of the attendances identified as cases of STR in the ED received an ICD-10 hospital discharge diagnosis categorized under ICD-10 group I63. Further common ICD-10 diagnoses were G45 (12.6 %), I61 (6.9 %), G40 (2.4 %), and I60 (2.1 %) (Table S4 in Multimedia Appendix 1).

For all 3 syndrome definitions, specificity and accuracy were high with over 94%. For the MI syndrome definition, we even calculated specificity and accuracy values at around 99%. For the ACS syndrome definition, we calculated a sensitivity of 85.3% (95% CI 84.7%-85.9%) and for MI of 56.6% (95% CI 55.1%-58.0%). The STR syndrome definition resulted in a sensitivity of 80.5% (95% CI 79.9%-81.2%) (Table 2).

**Table 2.** Sensitivity, specificity, and accuracy for the 3 syndrome definitions acute coronary syndrome (ACS), myocardial infarction (MI), and stroke (STR) in %, including 95% CI.

	Sensitivity, % (95% CI)	Specificity, % (95% CI)	Accuracy, % (95% CI)
ACS	84.4 (83.6‐85.2)	95.0 (94.7‐95.0)	94.7 (94.6‐94.8)
MI	56.6 (55.1‐58.0)	99.6 (99.6‐99.7)	99.0 (99.0‐99.0)
STR	80.5 (79.9‐81.2)	97.5 (97.5‐97.6)	96.7 (96.7‐96.8)

**Figure 4.**
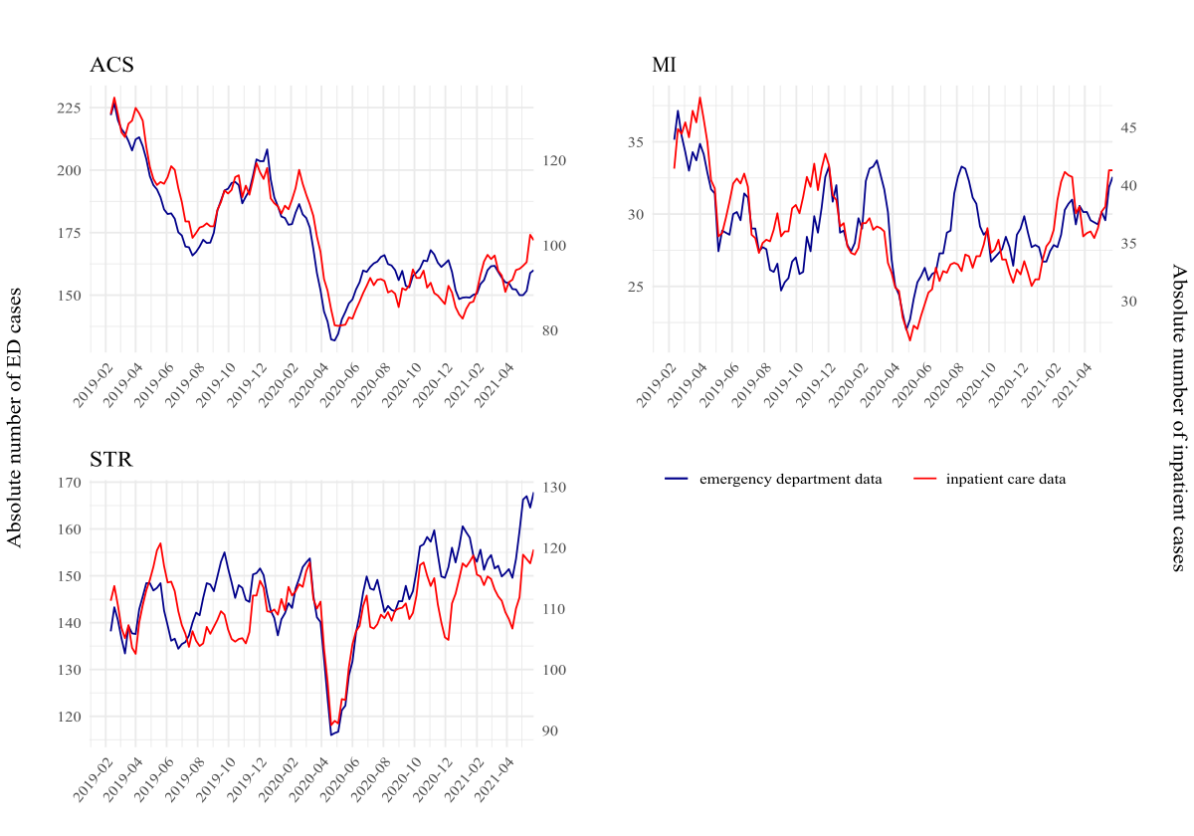
Time series showing a 7-day-moving average of the absolute number of cases of acute coronary syndrome (ACS), myocardial infarction (MI), and stroke (STR) identified in emergency department (ED) data (red, left *y*-axis) compared to inpatient care data (blue, right *y*-axis); Note: *y*-axes are not equal.

Sensitivity and specificity did not vary to a great extent over time for all 3 syndrome definitions (Figure S1 in Multimedia Appendix 1). When comparing ED cases with respective indicators from the hospital discharge data over time, both time series showed fairly similar patterns for all 3 indicators, particularly ACS and STR. The MI syndrome definition captured 2 peaks in March and August 2020, which were not evident in the hospital discharge data. Across all 3 indicators, a decrease in case numbers was observed between March and April 2020, aligned with the onset of the first wave of the SARS-CoV-2-pandemic (Figure 4).

### External Validation

**Figure 5.**
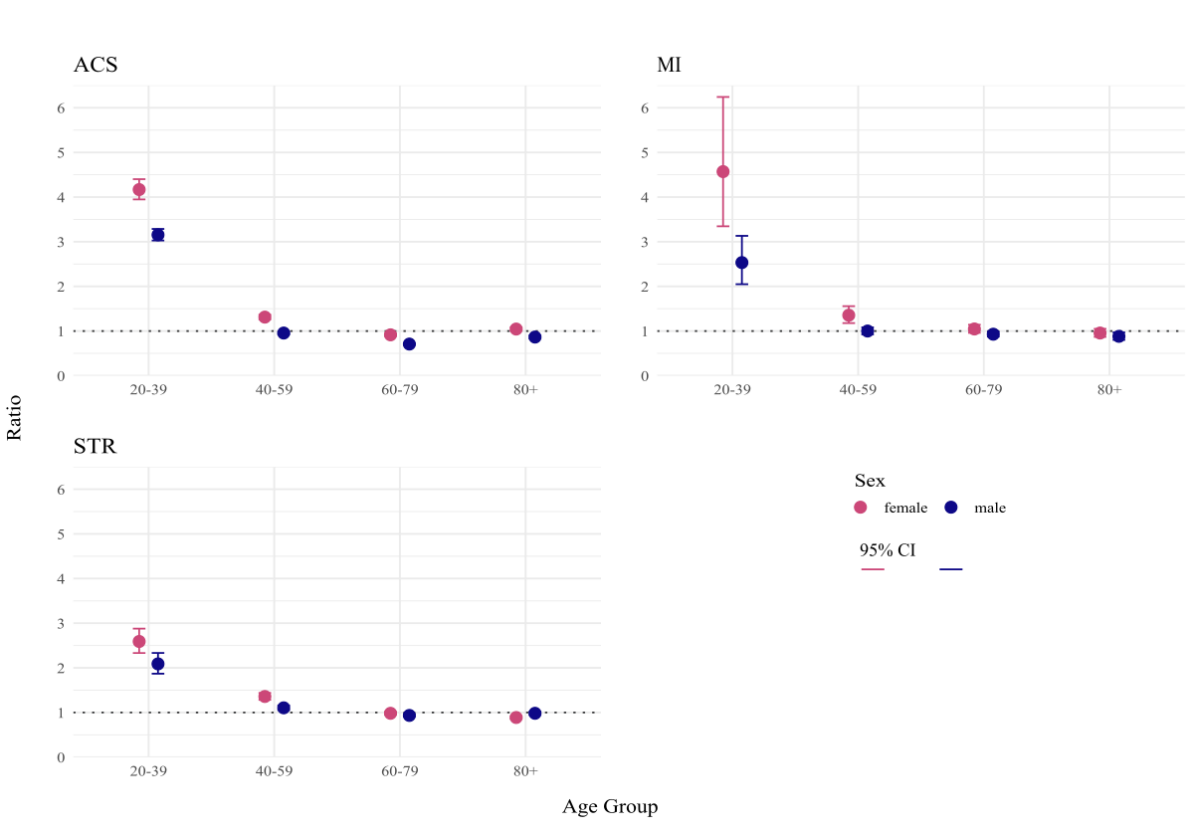
Ratios and 95% CIs calculated based on the comparison of age and sex stratified relative numbers of emergency department (ED) cases of acute coronary syndrome (ACS), myocardial infarction (MI), and stroke (STR) with data from the hospital diagnosis statistic.

Comparing cases in the ED for the years 2019 and 2020 with cases in the hospital diagnosis statistic, for all 3 syndrome definitions, ratios tended to be closer to one for the older age groups (Figure 5). For ACS, we calculated a ratio of 1.04 (95% CI 1.01‐1.08) for women aged 80 years or older and a ratio of 0.95 (95% CI 0.93‐0.99) for men between 40 and 59 years of age. For MI, comparing the relative numbers of men in the same age group resulted in a ratio of 1.00 (95% CI 0.92‐1.08). For the syndrome definition of STR, relative case numbers of men older than 80 years of age and women between 60 and 79 were highly comparable to the hospital diagnosis statistic, both with a ratio of 0.98. The biggest relative differences were observed in women of the age group of 20-39 years, where we calculated ratios of 4.17 (95% CI 3.94‐4.40) for ACS, 4.57 (95% CI 3.35‐6.24) for MI, and 2.59 (95% CI 2.33‐2.88) for the STR syndrome definition (Table S5 in Multimedia Appendix 1).

## Discussion

This study aimed to develop and validate syndrome definitions for identifying cases of ACS, MI, and STR in routine ED data. Syndrome definitions were created based on iterative discussions with experts from different fields. The detection of ACS and STR resulted in high sensitivity values, while specificity remained over 95% for all 3 syndrome definitions. At 56.6%, the sensitivity for MI was lower, indicating that syndromic surveillance using routine data is not ideal to identify cases of MI in the ED. This is, however, not surprising given the clinical reality in EDs. MI patients may generally present with an unspecific spectrum of complaints like shortness of breath, chest pain, or respiratory arrest. A confirmed diagnosis might then only be possible following a hospitalization. This is supported by another study conducted in the same setting, where a sensitivity of 48% was calculated for MI in the ED [26]. The ACS syndrome definition, on the other hand, is combining a broader range of complaints, including patients with MI but also, for example, angina pectoris or heart failure. When looking at the ICD-10 hospital discharge diagnoses for those cases, most received the relatively unspecific diagnosis code R07, which was surprising given our expectation that patients would be diagnosed more specifically once they were hospitalized. However, this result goes in line with a study conducted in one Berlin ED that listed the top 10 hospital discharge diagnoses for patients presenting with chest pain; while the relative proportions differ from our results, the 3 most common ICD-10 diagnoses identified by this study were also I20, I21, and R07 [27]. One hypothesis to explain the high number of unspecific discharge diagnoses could be insufficient documentation quality in the EDs, especially for outpatients. Another assumption is that especially the common and unspecific R07 diagnosis is used after all other urgent conditions have already been ruled out. In both cases, patients who did not receive a specific cardiovascular diagnosis during inpatient treatment do not fit the profile of cardiovascular emergencies that the ACS syndrome definition is supposed to detect.

When looking at the comparison of ED cases and hospital discharge diagnoses over time, we identified 2 peaks in the MI ED cases in March and August 2020. This correlates with the first and second waves of the COVID-19 pandemic in Germany [28]. Those differences might be due to changes in ED utilization during the pandemic. On the other hand, this possible overestimation of MI cases could be a result of COVID-19 cases presenting with unspecific symptoms diagnosed as MIs in the ED.

All 3 syndrome definitions demonstrated a high level of correspondence in age and sex distribution between ED data and hospital diagnosis statistics in the older age groups. We identified a higher proportion of cases between 20 and 39 years of age in the ED; the highest difference could be observed for women who were diagnosed with MI with our syndrome definition.

While the presented analyses outlined the validity of the created syndrome definitions, several limitations must be considered. First, we were only able to conduct our analyses based on a small subset of the available ED data, as we only included EDs with sufficient data quality in the overall analyses and restricted the internal validation to patients who were hospitalized (ie, attendances that received both an ED diagnosis and a hospital discharge diagnosis). However, the here-analyzed medical conditions are of high urgency and typically lead to hospitalization, which is why we assume our syndrome definitions to be applicable to other EDs. By linking the ED and inpatient care data on a case-based level, we were furthermore able to compare each attendance with their own hospital discharge diagnosis. Therefore, we assume the calculated sensitivity and specificity values to be reliable nevertheless. Another limitation might result from choosing our reference data for the sensitivity and specificity calculations. Hospital discharge diagnoses are coded at the very end of the patient’s treatment pathway and are therefore considered more valid than ED diagnoses [29]. They might, however, still be subject to misdiagnosis, as billing and other administrative processes can potentially introduce a bias. Nevertheless, German hospital discharge diagnoses are widely recognized as valid due to mandatory standard coding guidelines [29] and were therefore defined as the gold standard. Hence, we consider it unlikely that misdiagnosis to the extent where the ICD-10 code refers to an entirely different clinical presentation would occur at a rate that would substantially alter our results. The routine nature of the data leads to a limited flexibility regarding aggregation levels of certain values. Age, for example, is only available in 5-year age groups. For this reason, we had to include a limit of at least 20 years of age in our syndrome definitions, as opposed to using a more conventional cutoff of 18 years of age to differentiate from children and young adults.

The AKTIN registry does not have full coverage of all German EDs. The selection of EDs into the registry and, therefore, into the study is based on voluntary participation and might not be representative for the whole country. Nevertheless, comparing the age and sex distribution within the ED cases with all recorded diagnoses in Germany (external validation) strengthened our trust in the ability of our ED surveillance system to monitor changes of ACS, MI, and STR attendances, even with a small number of EDs.

The nature of the data itself leads to another caveat, especially when interpreting the absolute and relative number of cases identified through the syndrome definition. We rely on routine patient data not specifically collected for research or surveillance purposes. Therefore, data quality in general, as well as for specific values, may vary. For instance, there may be inconsistencies in the completeness of diagnoses and chief complaint information across different EDs; coding practices may differ between and within EDs and hospitals. These factors could lead to either over- or underestimating cases for all 3 syndrome definitions, posing challenges, particularly when reporting the absolute number of cases. To address this limitation in our routine surveillance, we chose to report only relative case numbers, using only attendances without missing values to calculate the denominator. It is crucial to emphasize that precise labeling of every single case is not essential for valid and reliable surveillance. The aim is to detect overall trends and changes in frequencies. While achieving high precision of a syndrome definition is beneficial, the results remain meaningful for public health despite some degree of variability.

In summary, we developed syndrome definitions to identify cases of ACS, MI, and STR in routine ED data. We validated them by comparing ED cases with those identified in hospital data. The calculated sensitivity, specificity, and accuracy measures demonstrate that the syndrome definitions are sufficiently valid to support continuous and timely surveillance of these health events. Integrating these indicators in our daily ED surveillance at the RKI would provide ad-hoc insights and time trends for ACS, MI, and STR, and thus enable more targeted public health actions and enhance the establishment of comprehensive public health surveillance in Germany.

## Supplementary material

10.2196/66218 Multimedia Appendix 1 Supplementary figures and tables.

## Abbreviations

ACS: acute coronary syndrome
CEDIS-PCL: Canadian Emergency Department Information System–Presenting Complaint List
CVA: cerebrovascular accident
ED: emergency department
GDPR: General Data Protection Regulation
ICD10-GM: International Statistical Classification of Diseases and Related Health Problems 10th Revision-German Modification
MI: myocardial Infarction
NCD: noncommunicable disease
RKI: Robert Koch Institute
STR: stroke
