# Supplementary material for: Establishing Syndromic Surveillance of Acute Coronary Syndrome, Myocardial Infarction, and Stroke: Registry Study Based on Routine Data From German Emergency Departments
Source: JMIR Public Health Surveill. 2025 Feb 25;11:e66218. doi: 10.2196/66218 (PMC11878330; doi:10.2196/66218)
Supplement: Multimedia Appendix 1 [file publichealth-v11-e66218-s001.docx]

Supplementary Table 1 Baseline table of ED attendances that could be linked to hospital discharge diagnosis by sex, age and triage level, as well as stratified by the syndrome definitions ACS, MI and STR

Supplementary Table 2 ICD-10 hospital discharge diagnoses groups for cases identified with ACS in the emergency department (Top 10 out of 450 diagnosis groups)

| **ICD-10 Diagnoses** | **N** | **%** |
| --- | --- | --- |
| R07 Pain in throat and chest | 4,283 | 19.7 |
| I21 Acute myocardial infarction | 3,837 | 17.6 |
| I20 Angina pectoris | 2,897 | 13.3 |
| I50 Heart failure | 1,395 | 6.4 |
| I48 Atrial fibrillation and flutter | 1,315 | 6.0 |
| I10 Essential (primary) hypertension | 1,101 | 5.1 |
| I26 Pulmonary embolism | 379 | 1.7 |
| I25 Chronic ischaemic heart disease | 316 | 1.5 |
| I47 Paroxysmal tachycardia | 294 | 1.4 |
| I49 Other cardiac arrhythmias | 238 | 1.1 |

Supplementary Table 3 - ICD-10 discharge diagnoses groups for cases identified with MI in the emergency department (Top 10 out of 398 diagnosis groups)

| **ICD-10 Diagnoses** | **N** | **%** |
| --- | --- | --- |
| I21 Acute myocardial infarction | 2,609 | 70.3 |
| I20 Angina pectoris | 158 | 4.3 |
| R07 Pain in throat and chest | 149 | 4.0 |
| I50 Heart failure | 112 | 3.0 |
| I10 Essential (primary) hypertension | 85 | 2.3 |
| I25 Chronic ischaemic heart disease | 67 | 1.8 |
| I48 Atrial fibrillation and flutter | 65 | 1.8 |
| R55 Syncope and collapse | 40 | 1.1 |
| I42 Cardiomyopathy | 33 | 0.9 |
| I11 Hypertensive heart disease | 22 | 0.6 |

Supplementary Table 4 - ICD-10 discharge diagnoses groups for cases identified with STR in the emergency department (Top 10 out of 358 diagnosis groups)

| **ICD-10 Diagnoses** | **N** | **%** |
| --- | --- | --- |
| I63 Cerebral infarction | 9,339 | 50.7 |
| G45 Transient cerebral ischaemic attacks and related syndromes | 2,318 | 12.6 |
| I61 Intracerebral haemorrhage | 1,269 | 6.9 |
| G40 Epilepsy | 450 | 2.4 |
| I60 Subarachnoid haemorrhage | 383 | 2.1 |
| H81 Disorders of vestibular function | 316 | 1.7 |
| H81 Disorders of vestibular function | 259 | 1.4 |
| G43 Migraine | 197 | 1.1 |
| S06 Intracranial injury | 187 | 1.0 |
| I64 Stroke, not specified as haemorrhage or infarction | 171 | 0.9 |

Supplementary Table 5 – Absolute numbers of cases of ACS, MI and STR in the ED data and hospital diagnosis statistic, as well as ratios and 95% confidence intervals calculated based on the comparison of age and sex stratified relative numbers of ED cases of ACS, MI and STR with data from the hospital diagnosis statistic

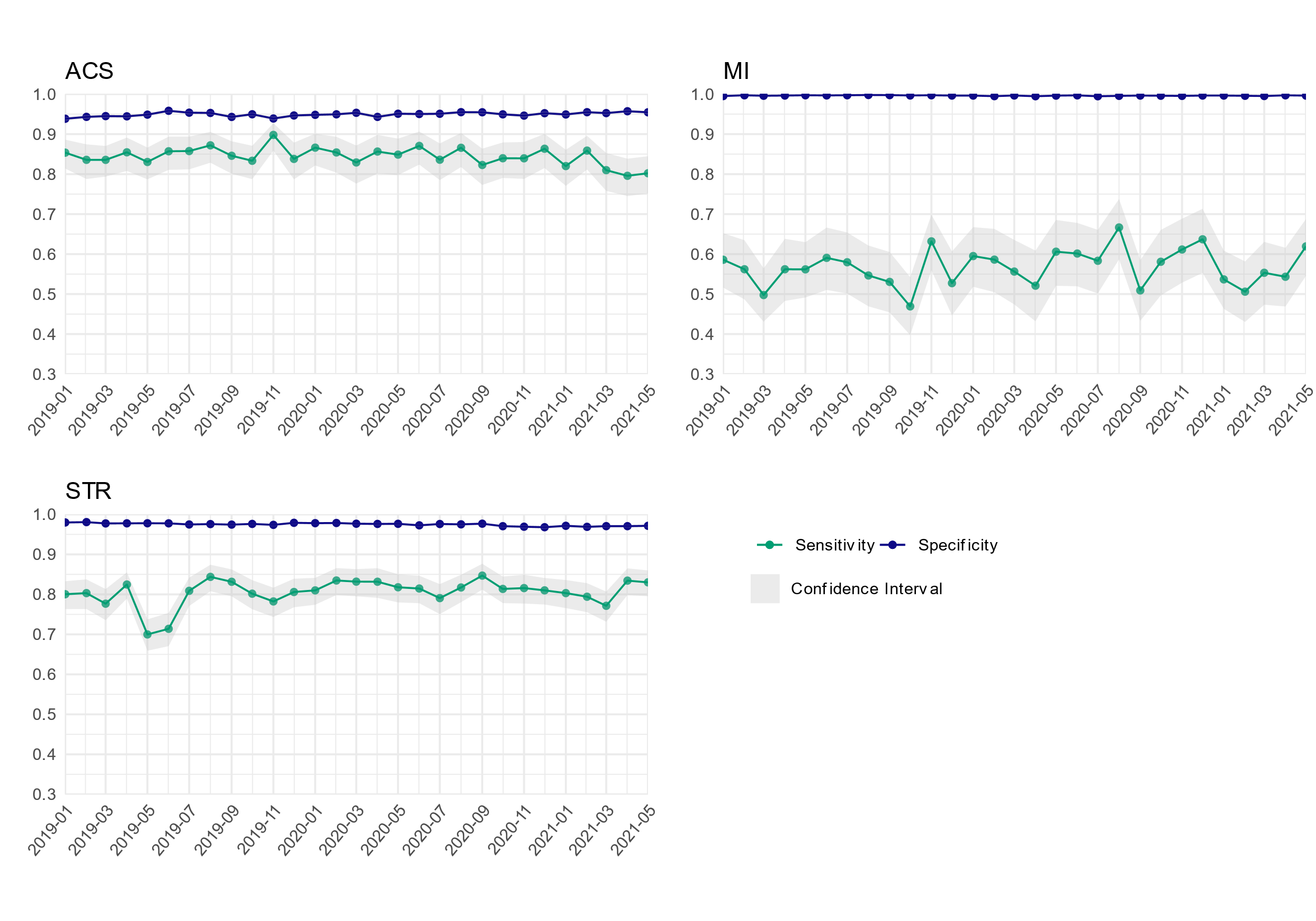


Supplementary Figure 1 – Sensitivity, specificity and 95% confidence interval per month for ACS, MI and STR
